# Utilizing genome-scale models to optimize nutrient supply for sustained algal growth and lipid productivity

**DOI:** 10.1038/s41540-019-0110-7

**Published:** 2019-09-24

**Authors:** Chien-Ting Li, Jacob Yelsky, Yiqun Chen, Cristal Zuñiga, Richard Eng, Liqun Jiang, Alison Shapiro, Kai-Wen Huang, Karsten Zengler, Michael J. Betenbaugh

**Affiliations:** 1 Department of Chemical and Biomolecular Engineering, Johns Hopkins University, 3400 North Charles Street, Baltimore, MD 21218 USA; 2 Department of Pediatrics, University of California, San Diego, 9500 Gilman Drive, La Jolla, CA 92093-0760 USA; 3 Department of Bioengineering, University of California, San Diego, 9500 Gilman Drive, La Jolla, CA 92093-0412 USA; 4 School of Environmental Science and Engineering, Shandong University, No.27 Shanda Nan Road, Jinan, 250100 China; 5 Center for Microbiome Innovation, University of California, San Diego, 9500 Gilman Drive, La Jolla, CA 92093-0436 USA

**Keywords:** Systems biology, Biotechnology, Computer modelling

## Abstract

Nutrient availability is critical for growth of algae and other microbes used for generating valuable biochemical products. Determining the optimal levels of nutrient supplies to cultures can eliminate feeding of excess nutrients, lowering production costs and reducing nutrient pollution into the environment. With the advent of omics and bioinformatics methods, it is now possible to construct genome-scale models that accurately describe the metabolism of microorganisms. In this study, a genome-scale model of the green alga *Chlorella vulgaris* (*i*CZ946) was applied to predict feeding of multiple nutrients, including nitrate and glucose, under both autotrophic and heterotrophic conditions. The objective function was changed from optimizing growth to instead minimizing nitrate and glucose uptake rates, enabling predictions of feed rates for these nutrients. The metabolic model control (MMC) algorithm was validated for autotrophic growth, saving 18% nitrate while sustaining algal growth. Additionally, we obtained similar growth profiles by simultaneously controlling glucose and nitrate supplies under heterotrophic conditions for both high and low levels of glucose and nitrate. Finally, the nitrate supply was controlled in order to retain protein and chlorophyll synthesis, albeit at a lower rate, under nitrogen-limiting conditions. This model-driven cultivation strategy doubled the total volumetric yield of biomass, increased fatty acid methyl ester (FAME) yield by 61%, and enhanced lutein yield nearly 3 fold compared to nitrogen starvation. This study introduces a control methodology that integrates omics data and genome-scale models in order to optimize nutrient supplies based on the metabolic state of algal cells in different nutrient environments. This approach could transform bioprocessing control into a systems biology-based paradigm suitable for a wide range of species in order to limit nutrient inputs, reduce processing costs, and optimize biomanufacturing for the next generation of desirable biotechnology products.

## Genome-scale models as a tool to optimize nutrient supply for different conditions

Supplying the optimal levels of nutrients such as nitrogen and carbon is an important consideration when cultivating green algae and other biological systems. Overfeeding nutrients can increase production costs while insufficient supplies can lower product yields. Finding the balance between production costs and productivity is a major challenge in biomanufacturing. Our published *C. vulgaris* genome-scale model was used to control the supplies of nitrate for algal growth under autotrophic or photosynthetic conditions, saving 18% of nitrate fed. Under heterotrophic conditions with organic carbon supplied, we optimized feeding of both glucose and nitrate while maintaining robust growth. The model was also used to enhance fatty acid volumetric product yields by over 60% and lutein levels nearly 3 fold under nitrogen-limited conditions. This study demonstrates that metabolic models can be used to predict the optimal nutrient supply as we strive to make algal bioprocessing sustainable for the future.

## Introduction

Microalgae offer significant opportunities to generate diverse products across many different areas including health products like lutein,^1^ pharmaceutical products like camelid heavy chain only antibodies (V_H_H),^2^ and energy precursors for biodiesel.^3^ Due to its high growth rate and high lipid content, *Chlorella vulgaris* has been recognized as a promising candidate for algal biofuel production.^4^ Furthermore, studies have found higher lipid productivity in *C. vulgaris* under heterotrophic growth conditions or nitrogen starvation.^4,5^ However, the cost of inputs including the source of nitrogen, often in the form of nitrate, as well as the organic carbon source, typically glucose in heterotrophic condition, can have a significant impact on overall productions costs.^6^ In addition, the reduction of growth rate under nitrogen starvation can represent another bottleneck due to the loss of algal biomass.

One approach to overcome these limitations is to optimize the natural cells growth and lipid production by controlling nutrient feeding. For example, one approach is to control glucose concentration within a defined range during algal culture using measurements and simple feedback control algorithms. Previous studies controlled glucose concentration in the range of 5 to 40 g/L to reach a high cell density culture, increasing cell density from 6.25 to 117.18 gDW/L in 32 h.^7^

In order to more effectively control nonlinear biological processes like cell culture, model predictive control (MPC), a model-based control strategy, has been designed as one approach to achieve more finely tuned bioreactor control.^8^ Mathematical equations can be constructed to represent the bioreactor system and nutrient supplies optimized based on the simulations. This strategy has been validated *in silico* for penicillin fermentation process, biohydrogen production in *Cyanothece sp*, and biofuel production in algae.^9–11^ It was also tested in *Chlorella* in a CSTR system for CO_2_ mitigation and in an open pond system under different light intensities.^12,13^ These MPC approaches typically incorporate empirical kinetic models based on experimental data in order to predict optimal algal growth conditions. Researchers obtain model parameters from experimental measurements of cell growth rate and nutrient consumption rate and construct equations in order to simulate algal growth in the models. Those parameters are often associated with the bioreactor system including volume and dilution rate or reflect external characteristics of the cell including growth rate.

However, these previous efforts typically lack a detailed description of the metabolic characteristics of the algal cells themselves and therefore cannot account for nutrient requirements and accompanying cell compositions. These models, while highly descriptive of growth at the macroscopic level, typically do not include the mathematical framework to describe exactly how specific nutrients such as nitrate and glucose are incorporated within the cellular metabolic framework under different culture conditions. Incorporating cellular metabolic details can enable biotechnologies to more effectively understand and potentially optimize the utilization of nutrients within algal cells during growth and lipid production, and be able to consider changes in algal metabolism for different nutrient input scenarios.

Fortunately, the advent of genome-scale models has enabled the biotechnology community to better understand the allocation and distribution of nutrients to metabolites and biomass.^14^ Genome-scale metabolic models are a system biology tool that represent the metabolism of a cell based on its genomic sequence. In order to reconstruct biological networks into a genome-scale model, metabolic reactions in the form of mass balance equations that integrate nutrient transport, intracellular metabolism, and biomass accumulation are included.^15^ Model constraints are obtained by considering genomics, transcriptomics, proteomics as well as cellular composition information.^16^ Thousands of metabolic reactions are composed as a large stoichiometric matrix (*m* x *n*); followed by flux balance analysis required that is solved in order to identify the flux rates of each reaction. Furthermore, as the model is typically underdetermined, linear programming is incorporated in order to identify an optimal flux solution by maximizing an objective function, typically the biomass production rate.^17^

Genome-scale models have been constructed for a wide variety of industrial relevant species including *E. coli*,^18^ yeast^19^ and mammalian cells.^20^ Indeed, previous research has applied genome-scale models to a variety of applications including microbial strain optimization,^21^ intracellular metabolite pool prediction,^16^ and the discovery of new metabolic reactions.^22^ Recently, our group constructed a genome-scale model for *C. vulgaris* (*i*CZ843), which provides the most comprehensive representation of the physiology of this organism to date, including 843 genes, 2,294 reactions, and 1,770 metabolites.^23^ This model contains five cellular compartments including the cytoplasm, mitochondrion, chloroplast, thylakoid and glyoxysome, in addition to the extracellular environment, allowing metabolic exchange within and between the different compartments. In addition, corresponding experiments successfully validated model predictions, including the capacity to vary the growth rate of *C. vulgaris* by altering nutrient inputs. Furthermore, the *i*CZ843 model was used to contextualize metabolomics data over the course of growth, evaluating dynamic changes in the biomass composition under different nutrient conditions, resulting in an updated model of *C. vulgaris* model (*i*CZ946).^16^

In genome-scale model studies, biomass production is typically set as the objective function in order to drive the optimization in the model and predict cell growth rates.^17^ However, for our metabolic model control (MMC) applications, the primary objective may be the capacity to control nutrient utilizations in order to control inputs that can then be provided based on model predictions. In the current study, growth of *C. vulgaris* was estimated based on experimental optical density (OD) measurements and then used to predict subsequent growth rates during the exponential phase. Since the primary objective of this study was to optimize the nutrient supply for algal culture, the objective function was instead changed to optimize nutrient flux input, including nitrate or glucose uptake into algal cells. We then applied the genome-scale model in order to improve predictions of the nutrients required for growth in response to different culture conditions. As a result, we demonstrate the capacity to utilize the metabolic model control in order to more effectively and efficiently optimize bioprocessing for the generation of high levels of algal biomass and biofuel precursors for potential applications in biotechnology. More importantly, this framework demonstrates a new avenue for applying genome-scale models as a way to optimize bioprocessing across a wide range of cell lines and potential applications in biotechnology.

## Results

### Controlling nitrate addition under autotrophic condition for optimized growth rates

**Fig. 1:**
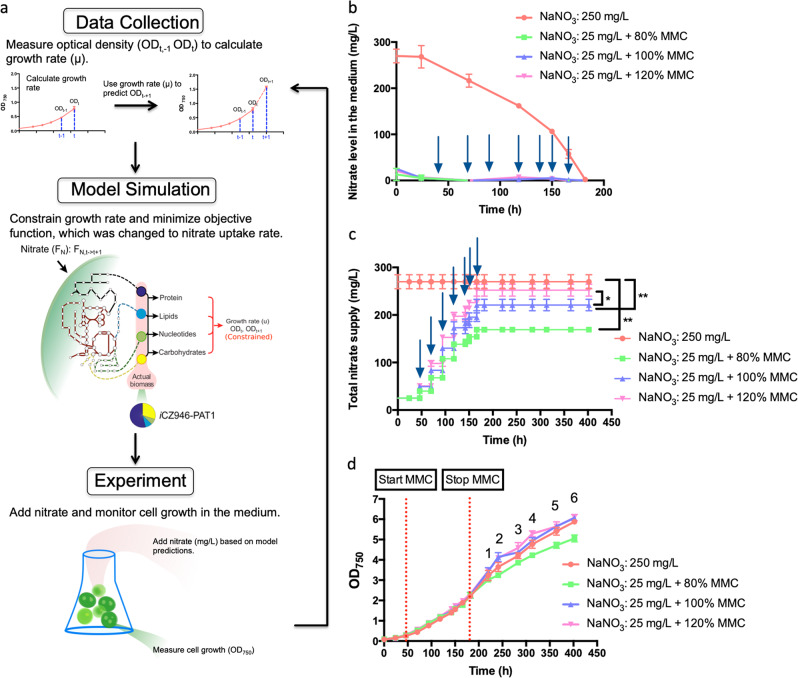
Metabolic model control under autotrophic conditions. Arrows indicate the feeding time points; **a** Algorithm of metabolic model control (MMC); **b** Nitrate level in the medium (mg/L); **c** Total nitrate supply during the culture (mg/L); **d** Growth curve (OD_750_). The *p*-value at time points 1–6 was provided in Table S2. The data represents the mean ± SD for *n* = 3. *P ≤ 0.05 **P ≤ 0.01

In order to evaluate the MMC approach, model *i*CZ946-PAT1 was used to control nitrate supply to autotrophic *C. vulgaris* cultures. Model *i*CZ946-PAT1 was previously constructed based on the biomass composition obtained under phototrophic and nitrogen sufficient conditions (Fig. S1 and Table S1). In the current approach, the growth rate (μ) was calculated based on the optical density value (OD_750_) taken from experimental data at a previous time point (OD_t-1_) and the current time point (OD_t_). Then, the growth rate within the model was constrained to the calculated growth value (μ) and the objective function was changed to the nitrate uptake rate (F_N_). Specifically, the model was optimized to calculate the minimum nitrate amount required to sustain this calculated growth rate over a particular time interval (Fig. 1a).

One uncontrolled *C. vulgaris* culture was cultured with 250 mg/L nitrate, while three others were cultured with 25 mg/L nitrate and fed with 80%, 100 and 120% of the optimal nitrate requirement based on model predictions after 25 mg/L nitrate was consumed completely in the medium. For example, if the genome-scale model predicted *C. vulgaris* cultures need 100 mg/L nitrate to sustain the growth for the next 24 h, we would add 80 mg/L, 100 mg/L, and 120 mg/L to MMC-80%, MMC-100%, and MMC-120% cultures respectively. Based on the culture volume, a specific amount of nitrate was fed to the culture to reach the concentration predicted by the model. All cultures were bubbled with 5% CO_2_ in order to make excess carbon source available in culture and therefore make nitrate the main variable affecting the growth rate. The controlled cultures fed with nitrate at 25 mg/L consumed all the nitrogen source by 46 h. Genome-scale metabolic model control was then applied to calculate the subsequent nitrate supply needed at 46, 70, 94, 118, 142, 150, and 166 h (see arrows) (Fig. 1a, b). Eventually, the uncontrolled *C. vulgaris* cultures consumed all 250 mg/L nitrate by 182 h and the model predictive feeding was stopped after that time point. Measured nitrate concentration was nearly 0 mg/L in the medium at all the time points after 46 h for all MMC conditions, indicating that the total nitrate supplied to the medium was transported or consumed completely into the cell.

Nitrate was added to all the three MMC cultures at 46, 70, 94, 118, 142, 150, and 166 h to reach a total amount available of 170 mg/L, 220 mg/L, and 250 mg/L over the entire duration for the 80%, 100%, and 120% MMC conditions, respectively (Fig. 1C). Even though significantly <250 mg/L nitrate was fed to the 80% and 100% MMC cultures (*P* ≤ 0.05), growth rates comparable to the bulk-fed group were achieved in the 100% MMC cultures (Fig. 1d). All four cultures reached OD_750_ = 2.2 in 182 h, after which algae entered a nitrogen starvation stage when the MMC feeding was stopped. As displayed by the 120% MMC condition, even though cells took up all of the nitrate supplied to the medium, feeding an amount of nitrate higher than predicted by the genome-scale model did not benefit algal growth in nitrogen replete or nitrogen depleted conditions (Fig. 1d). This result was further supported by the growth rate obtained for the bulk-fed 250 mg/L, which was similar to that obtained for 100% MMC culture. Even though the bulk-fed and the MMC 120% group consumed 22% and 14.5% more nitrate than the 100% MMC group respectively, all three groups exhibited nearly comparable growth rates (Table S2, Fig. 1c, d). However, supplying 80% of the nitrate amount predicted by the model resulted in a slower growth rate especially after 241 h (*P* ≤ 0.05) (Fig. 1d and Table S2). The slow growth rate of the 80% MMC group and unimproved growth rate of 120% MMC group indicate that the genome-scale model can predict the optimal nitrate amount required for *C. vulgaris* growth.

In addition, we also performed an experiment to show that by incorporating real-time measurements, this MMC approach help to enhance the performance of algal cultures and also control nutrient supply more efficiently compared with standard approaches based on biological approaches.

In this experiment, *C. vulgaris* culture was fed with nitrate using 3 different feeding strategies. The control case included an initial loading of 285 mg/L nitrate with no subsequent feeding. A second case was based on the biological knowledge of the growth rate (0.0137 h^−1^) from the previous experiment (Fig. 1) to predict the nitrate supply; this nitrate was fed every 12 h, which was actually more often than the MMC case followed (Fig. S2a). For the third MMC conditions, however, we measured the OD_750_ value every 24 h, and then input the growth rates into our genome-scale model to determine the proper amount to feed every 24 h for nitrate control in the MMC cultures (Fig. S2a).

The uncontrolled *C. vulgaris* culture was fed with 285 mg/L nitrate initially and the nitrate was consumed completely in the media by around 147 h (Fig. S2b). For the biological based feeding case, the OD_750_ reached a slightly lower value of 3.9 while also utilizing approximately 285 mg/L nitrate by experimental completion. However we added a total of only around 233 mg/L nitrate to the MMC cultures saving ~18% nitrate while reaching a similar OD_750_ around 4.3 at 255 h as the uncontrolled batch experiment (Fig. S2c, d). By using real-time OD_750_ measurements, we can predict growth rate and supply nutrients more accurately.

### The growth rates of *C. vulgaris* can be controlled under heterotrophy

**Fig. 2:**
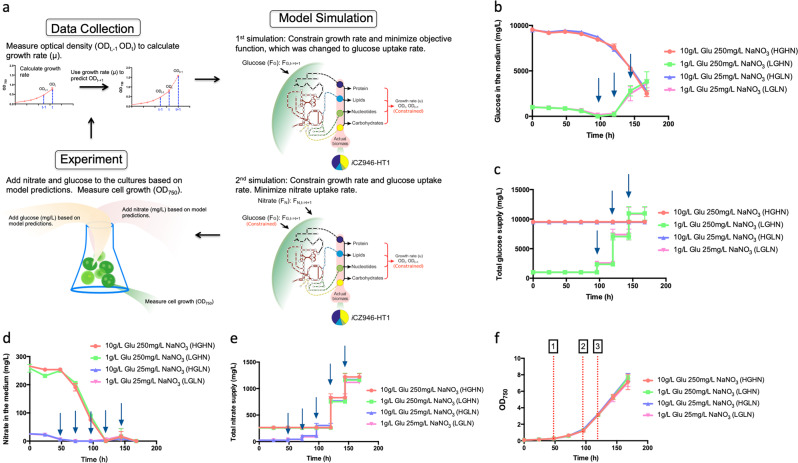
Metabolic model control under heterotrophic conditions without constraints on glucose uptake rate. Arrows indicate the time points for feeding nutrients. **a** Algorithm of metabolic model control; **b** Glucose level in the medium (mg/L); **c** Total glucose supply during the culture (mg/L); **d** Nitrate level in the medium (mg/L); **e** Total nitrate supply during the culture (mg/L); **f** Growth curve (OD_750_). Point 1: 25 mg/L nitrate run out; Point 2: 1 g/L glucose run out; Point 3: 250 mg/L nitrate run out. The data represents the mean ± SD for *n* = 3

After successfully applying the genome-scale model *i*CZ946-PAT1 to control nitrate utilization under autotrophy, a new glucose control feature was included in the model to simulate heterotrophic growth. In this experiment, we applied the *i*CZ946-HT1 model, which used biomass composition and omics data experimentally determined under heterotrophic and nitrogen sufficient conditions (Fig. S1 and Table S1). By using this growth data, we evaluated the demand of glucose and nitrogen in order to characterize growth conditions. First, the measured optical density (OD_t−1_, OD_t_) was used to calculate the growth rate (μ) and to constrain biomass accumulation rate in *i*CZ946-HT1. Next, the glucose uptake rate (F_G_) was set as the objective function (Fig. 2a). The model was then optimized to calculate the minimum amount of glucose (F_G,t->t+1_) required to sustain algal growth. Then, glucose uptake rate was constrained using the predicted value and nitrate uptake rate (F_N_) was set as a new objective function in a subsequent simulation (Fig. 2a). The optimization then yields the amount of nitrate (F_N,t->t+1_) required to sustain algal growth.

This algorithm was performed at combinations of two different levels of glucose (10 g/L [HG], 1 g/L [LG]) and two different levels of nitrate (250 mg/L [HN], 25 mg/L [LN]). Experimental measurements showed that the low glucose groups (LGHN and LGLN) consumed all the initial glucose within 96 h (Fig. 2b). Based on the experimental culture volume, specific amounts of glucose were fed to the low glucose groups every 24 h based on experimental culture volume and model predictions (see arrows in Fig. 2b). The added glucose was nearly consumed between 96 and 120 h (Fig. 2b). However, after 120 h, the glucose levels started to increase in both LG groups, indicating the glucose was not completely consumed in the culture medium. At 168 h, ~3800 mg/L and 3600 mg/L of glucose was left in LGHN and LGLN groups, respectively (Fig. 2b). A total of around 11,000 mg/L was fed to both groups over 168 h (Fig. 2c), resulting in overfeeding after 120 h.

Experimental results indicated that *C. vulgaris* consumed 25 mg/L nitrate (HGLN and LGLN) and 250 mg/L nitrate (HGHN and LGHN) by 48 and 120 h. Specific amounts of nitrate were fed to the cultures every 24 h based on cell growth rate and model predictions for nitrogen uptake rate (Fig. 2d, e). Unlike the trends observed for glucose supplementation under the heterotrophic conditions, the algae consumed nearly all the nitrate added during each time interval between 48 and 168 h (Fig. 2d). In this way, the nitrate consumption pattern for *C. vulgaris* was different from the glucose uptake rate over the same period, in which glucose consumption rate did not match model predictions.

Biomass accumulation in all four *C. vulgaris* cultures was tracked as OD_750_ increased from 0.08 to 7.7 between 0 and 168 h (Fig. 2f). Initial low nitrate (25 mg/L) and low glucose (1 g/L) levels were exhausted at 48 (time point 1) and 96 h (time point 2), respectively, and the initial nitrate was exhausted at 120 h (time point 3), for the high nitrate case (250 mg/L). The uncoupling of glucose consumption and model predictions at 120 h suggests a potential bottleneck to glucose consumption at high cell densities of *C. vulgaris* (Fig. 2b), perhaps due to a limitation in oxygen supply or a limitation in glucose uptake rate that can occur at higher glucose levels.^24–26^ As a result, the current model for heterotrophic growth did not account for these observed bottlenecks to glucose consumption.

**Fig. 3:**
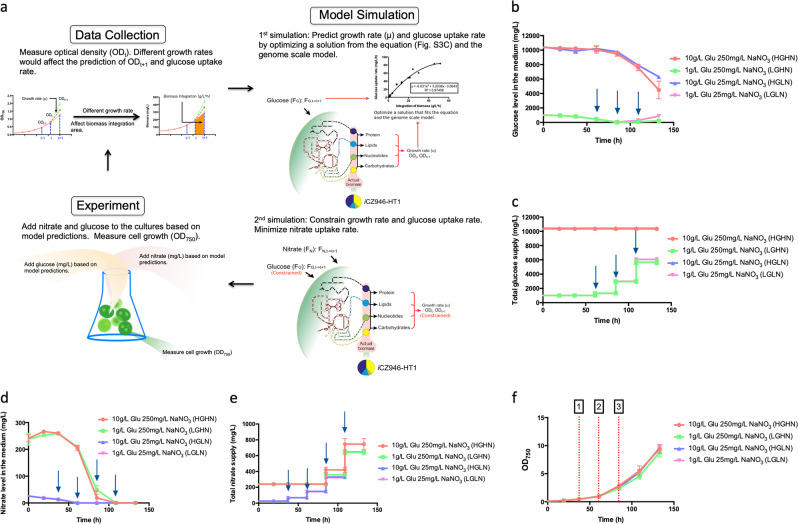
Metabolic model control in heterotrophic conditions with a constraint on glucose uptake rate. Arrows indicate the time points for feeding nutrients. **a** Algorithm of metabolic model control (MMC); **b** Glucose level in the medium (mg/L); **c** Total glucose supply during the culture (mg/L); **d** Nitrate level in the medium (mg/L); **e** Total nitrate supply during the culture (mg/L); **f** Growth curve (OD_750_). Point 1: 25 mg/L nitrate run out; Point 2: 1 g/L glucose run out; Point 3: 250 mg/L nitrate run out. The data represents the mean ± SD for *n* = 3

Therefore, the MMC algorithm was modified to reflect more accurately the reduced glucose uptake rate in algal culture occurring at higher biomass concentrations. Previously, glucose uptake limitations into *Chlorella* have been observed and modeled to account for substrate limitation.^24,26^ To account for such limitation in our studies, we obtained an empirical equation describing the relationship between glucose consumption rate and biomass build up rate and this equation was incorporated into the model. The relationship was determined using the integrated value of total biomass produced and glucose uptake rate (Fig. S3a, b). A polynomial regression line accurately fit the trend of glucose uptake rate as a function of biomass accumulation by using GraphPad software (*R*^2^ = 0.947) (Fig. S3c). This modification was incorporated into MMC to account for the limitation in the cellular glucose uptake rate capability (Fig. 3a). Consideration of both constraints restricts both the growth rate prediction and the glucose uptake rate. By utilizing this new equation to predict glucose uptake rate, we were able to estimate effective growth rates. As a result, we achieved more representative glucose and nitrate uptake rates.

Experimental validation was performed again with four different combinations (HGHN, LGHN, HGLN, and LGLN). *C. vulgaris* consumed all the initially fed glucose in the LG group (1 g/L; LGHN and LGLN) between 61 and 85 h (Fig. 3b). The MMC algorithm, was used to supply glucose after 61 h. Applying this new constraint on glucose uptake in the model dramatically decreased the amount of surplus glucose in the medium to 0–900 mg/L in the LG group (1 g/L; LGHN and LGLN) during the period of metabolic model control between 61 and 133 h. Around 5600–6000 mg/L glucose was fed to the LG group (1 g/L) during the same period of time (Fig. 3c). In the HG group (10 g/L; HGHN and HGLN), *C. vulgaris* only consumed 4100–5800 mg/L of the initially provided 10,000 mg/L glucose during the experiment, and left a significant amount of glucose in the medium (Fig. 3b).

Regarding the LN groups (25 mg/L; HGLN and LGLN), 25 mg/L of nitrate was consumed after 37 h and 250 mg/L of nitrate was consumed between 85 and 109 h for the HN groups (250 mg/L; HGHN and LGHN) (Fig. 3d). Nitrate control was started in the low nitrate group around 37 h and for the high nitrate group around 85 h. Between 640 mg/L and 740 mg/L of nitrate was fed to all four cultures (HGHN, HGLN, LGHN, LGLN) increasing OD_750_ from 0.08 to the range of 8.8 and 9.7 in 133 h (Fig. 3e, f).

### Modifying biomass composition in genome scale model to control nitrate supply for sustaining algal growth with high lipid productivity

Under nitrogen starvation, *C. vulgaris* has been found to accumulate high intracellular lipid levels by altering its biomass composition from low fatty acid content (~10%) to up to 60% fatty acid.^4^ *C. vulgaris* is able to accomplish this shift in biomass composition by recycling intracellular protein back into amino acids, which contributes to lipid synthesis.^27^ However, algae suffer two major drawbacks as a result of this adjustment which are the loss of protein and chlorophyll.^28^ These sacrifices of critical cellular components are likely to lead to an eventual cessation in growth. Indeed, we observed that *C*. *vulgaris* ceases growth during nitrogen starvation when protein content falls below ~10% (Fig. S4). Our genome scale model was therefore applied to control nitrate supply in order to sustain a protein level around 10% sufficient for continued algal growth even under stressed conditions.

**Fig. 4:**
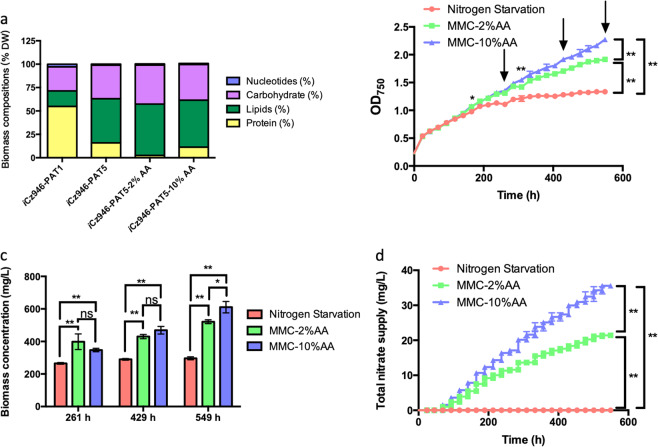
Metabolic model control under nitrogen limitation. **a** Biomass compositions in the models (normalized to 100%); **b** Growth curve (OD_750_); **c** Biomass concentration (mg/L); **d** Total nitrate supply during the culture (mg/L). The data represents the mean ± SD for *n* = 3. *P ≤ 0.05 **P ≤ 0.01

We previously explored the impact of different nutrient condition changes on the biomass composition and the metabolism of algal cells in a genome scale model.^16^ In the current work, the two different models, *i*CZ946-PAT1 and *i*CZ946-HT1, representing autotrophic and heterotrophic cultures, were incorporated into our metabolic model control algorithm and successfully validated the feasibility of nutrient optimization, as shown in Figs. 1 and 3. To account for these changes in algal biomass composition under nitrogen starvation, amino acid content was modified from the previously published autotrophic genome-scale model *i*CZ946-PAT5 (Table S1), which assumed 16% of amino acid content in the biomass composition and was constructed using omics data under nitrogen starvation conditions. Our modified models were constructed to include instead 10% amino acid content (*i*CZ946-PAT5-10%AA) and 2% amino acid content for the biomass composition (*i*CZ946-PAT5-2%AA), as shown in Fig. 4a. The nitrate amount calculated for *i*CZ946-PAT5-10%AA model should permit algae to synthesize enough protein and preserve sufficient chlorophyll in order to maintain steady algal growth while also potentially yielding a high lipid content under nitrogen starvation metabolism. In contrast, *C. vulgaris* was expected to cease growing when the nitrate supply was restricted to 2% as predicted by the *i*CZ946-PAT5-2%AA model.

To validate this concept, we again performed an autotrophic experiment in which nitrate was supplemented to cultures every 20–24 h based on our model prediction (Fig. 4b). One set of two flasks were subjected to nitrogen starvation and two other sets were supplied with a small amount of nitrate based on model predictions using either the *i*CZ946-PAT5-10% AA model or *i*CZ946-PAT5-2%AA model. For the first 165 h of growth, all *C. vulgaris* cultures grew similarly and reached OD_750_ = 1.0 (Fig. 4b). However, after 165 h, MMC cultures supplemented with nitrate started to grow faster than the nitrogen starvation cultures (*P* ≤ 0.05). At 261 h, *C. vulgaris* reached OD_750_ = 1.1 for the nitrogen starvation culture, while OD_750_ increased to 1.3 in both MMC-2%AA and MMC-10%AA cultures. At the end of the experiment, around 549 h, OD_750_ only increased slightly to 1.3 for the nitrogen starvation culture. In the MMC-2%AA case, algal growth progressively slowed relative to the 10%AA around 309 h (*P* ≤ 0.01), while still greater than the complete nitrogen starvation case. In contrast, the MMC-10%AA culture continued to expand over the complete duration of the culture period and reached a final OD_750_ of 2.3 at 549 h (*P* ≤ 0.01). These results indicated that an average of 0.06 mg/L/h nitrate could support algal growth while targeting a protein content at or near 10%.

Dry biomass was examined at three different time points: 261 h, 429 h, and 549 h, representing periods at which growth slowed significantly for the reduced and depleted nitrate cases (Fig. 4c). Indeed, the nitrogen starvation group, which stopped growing after around 237 h, showed similar biomass yields at its last two time points, ranging from 290 mg/L to 300 mg/L. The MMC-2%AA group showed an increase in biomass between all time points, growing from 390 mg/L at 261 h to ~430 mg/L at 429 h and 520 mg/L at 549 h. *C. vulgaris* maintained steady growth under stressed conditions for the MMC-10%AA condition. The biomass yield steadily increased from 340 mg/L at 261 h, to 470 mg/L at 429 h, finally reaching 610 mg/L at 549 h. The addition of nitrate using the *i*CZ946-PAT5-10%AA model facilitated a biomass increase of 30% at 261 h, 61% at 429 h, and 105% at 549 h compared to the complete nitrogen-starvation case (*P* ≤ 0.01). Compared with MMC-2%AA cultures, the biomass increased 17% at 549 h (*P* ≤ 0.05) with no significant difference at 261 h and 429 h (Fig. 4c). In total, around 36 mg/L and 21 mg/L nitrate were added to the MMC-10%AA and MMC-2%AA cultures respectively over the 549 h cell culture period (Fig. 4d). The reduction in growth rate after 309 h and biomass at 549 h in the MMC-2%AA cultures suggested that the supplied nitrate in MMC-2%AA condition was insufficient to maintain growth in the late stages of cultivation, confirming the need for a higher amino acid content in the model in order to maintain sufficient algal growth.

**Fig. 5:**
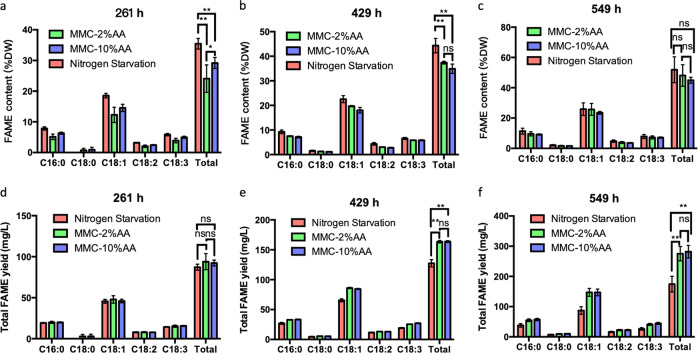
Fatty acid production at different time points. FAME content (% DW) at **a** 261 h, **b** 429 h, **c** 549 h. Total FAME yield (mg/L) at **d** 261 h, **e** 429 h, **f** 549 h. The data represents the mean ± SD for *n* = 3. *P ≤ 0.05 **P ≤ 0.01

FAME content and total FAME yield were measured to evaluate the role of using MMC on lipid productivity. As expected, at 261, 429 and 549 h, algae in the nitrogen starvation group accumulated the highest FAME content among the three different conditions: 35% at 261 h, 44% at 429 h and 52% at 549 h (Fig. 5a–c). Alternatively, at 549 h, the MMC-2%AA group accumulated 48% FAME content and the MMC-10%AA group accumulated 45% FAME content, which were 4% and 7%, below the nitrogen starvation case. This slightly decreased FAME content is to be expected, as a greater fraction of algae metabolism was dedicated to maintaining chlorophyll and protein content in the MMC cultures.

Importantly, the overall FAME yield, which incorporates both FAME content and biomass production, exhibited a different accumulation pattern. While the nitrogen starvation group yielded 175 mg/L at 549 h, the MMC-2%AA and MMC-10%AA group provided 275 mg/L and 281 mg/L total FAME yield respectively. Compared with MMC-10%AA conditions, the slower growth rate at 549 h did not affect the FAME yield in MMC-2% AA cultures. Over the three sampling periods, the MMC-10%AA group displayed an increasingly higher total FAME yield 28% and 61% when compared to the nitrogen starvation group at 429 h and 549 h (*P* ≤ 0.01), respectively.

### Change in biomass constituents under nitrogen starvation and metabolic model control conditions

**Fig. 6:**
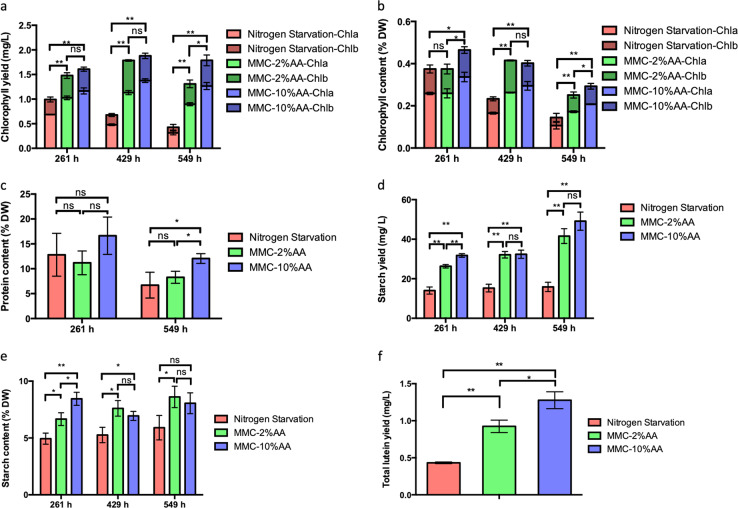
Biomass compositions during nitrogen limitation with metabolic model control. **a** Total chlorophyll yield (mg/L); **b** Chlorophyll content (% DW); **c** Protein content (% DW); **d** Total starch yield (mg/L); **e** Starch content (% DW); **f** Total lutein yield (mg/L) at 549 h. The data represents the mean ± SD for *n* = 3. *P ≤ 0.05 **P ≤ 0.01

After successfully controlling *C. vulgaris* growth with high FAME productivity under nitrogen starvation using our genome-scale metabolic model control approach, dry biomass was analyzed to compare the effect of MMC on other biomass constituents. Total chlorophyll yield decreased from 0.99 mg/L at 261 h to 0.43 mg/L at 549 h for the nitrogen deprivation case (Fig. 6a). Indeed, *C. vulgaris* is known to degrade the chlorophyll during nitrogen starvation conditions.^29^ Supplying insufficient levels of nitrate to the MMC-2%AA group also resulted in degradation of chlorophyll from 1.48 mg/L at 261 h to 1.31 mg/L at 549 h. Alternatively, total chlorophyll was maintained between 1.61 to 1.79 mg/L from 261 to 549 h in the MMC-10%AA group. Since overall biomass steadily increased for both MMC groups, the chlorophyll content decreased across all the cultures (Fig. 6b). However, the chlorophyll content was around 0.29% in MMC-10%AA cultures, higher than 0.25% in MMC-2%AA and 0.14% in nitrogen starvation (*P* ≤ 0.05). These results suggested that the *i*CZ946-PAT5-10%AA model can predict the level of nitrate necessary to maintain substantial chlorophyll content in *C. vulgaris* for longer periods, sustaining the energy capture capability from sunlight and building up biomass with an elevated fatty acid content in a limiting nitrogen environment.

Since nitrate is a major source of nitrogen for protein synthesis in algae, the protein contents were also measured for all three conditions. At 261 h, protein content for the nitrogen starvation, MMC-2%AA and MMC-10%AA cases were around 11–16% (Fig. 6c). By 549 h, protein content decreased to 6.7%, 8.2% and 12% under complete nitrogen starvation, MMC-2%AA and the MMC-10%AA conditions, respectively. We hypothesized that 10% protein may be the approximate lower threshold at which algae can maintain metabolic functions and continue growth under nitrogen limitations (See Fig. S4). Indeed, our experiment confirmed this lower threshold, as nitrogen starvation ceased growing around 165 h and MMC-2%AA groups growth progressively slowed around 309 h (Fig. 4b). Meanwhile, the protein content for the MMC-10%AA group was sustained around 10% even at 549 h. Previously, protein content has been found to decrease during nitrogen starvation,^30^ but this is the first study to show that continual supplementation of nitrate can maintain the protein content at a reduced level.

In addition to lipids, algae also use starch to store the energy captured from sunlight. Since more chlorophyll was present in the two MMC groups, more energy should be captured in the algal cells, therefore we also determined the starch yield and starch content in this work. We found that under nitrogen starvation conditions, total starch yield was constant between 14 and 15 mg/L between the three time points (Fig. 6d). In the two MMC groups, the total starch yield increased stably to a higher level than complete nitrate starvation at 261–549 h (*P* ≤ 0.01) with 42 mg/L and 49 mg/L for the MMC-2%AA and MMC-10%AA group. Previous studies with *C. zofingiensis* found that starch synthesis was more active during the initial stages of nitrogen starvation, but decreased gradually as nitrogen starvation continued.^31^ Our results indicated that without MMC, cells suppressed starch synthesis from 261 to 529 h. For both MMC groups, our results indicated that starch synthesis driven by sunlight was faster than the degradation due to limitations in the nitrogen levels. The starch content in both MMC groups was also higher than in nitrogen starvation case at most of the time points (Fig. 6e). Total lutein yield was also measured; using the *i*CZ946-PAT5–10%AA model resulted in enhanced lutein yields of 195% and 38%, when compared to the nitrogen starvation group (*P* ≤ 0.01) and the MMC-2%AA group at 549 h (*P* ≤ 0.05), respectively (Fig. 6f).

## Discussion

In this work, we demonstrate the application of a genome-scale model for metabolic model control for three different algal culture conditions. Under autotrophic conditions, we validated that this approach could provide an accurate prediction of the amount of nitrate needed without affecting cell growth, saving 18% of nitrate. Under heterotrophic conditions, we showed that this algorithm could also be applied to calculate multiple nutrient demands including glucose and nitrate while maintaining efficient growth. We also confirmed that this methodology could be applied to cultures under restricted nitrogen conditions. The model was used to predict a sufficient amount of nitrate needed to maintain algal growth rate while targeting a reduced protein content, enabling the cells to maintain fatty acid and lutein productivity. This MMC strategy led to a 61% increase in total FAME yield and 195% increase in total lutein yield at 549 h compared to the nitrate deprivation case.

Interestingly, we observed that even though a theoretical surplus of nitrate was fed for the 120% MMC condition according to model predictions, the cells still took up all the supplied nitrate experimentally under autotrophic conditions. Comparing the results with the 100% MMC condition suggested that this nitrate was not used to build more biomass. The phenomena of nitrate uptake rate exceeding nitrogen build up rate in biomass has been indicated previously with our genome-scale model.^16^ Also, Droop and coauthors previously described a phenomena of nitrogen source storage in order to account for algal growth after nitrate is exhausted in the medium.^10,32^ In our metabolic model control approach, nutrient control prediction is driven by growth rate and therefore this method reflects directly the proper nitrate amount needed to build up biomass. It is possible that some of the surplus nitrogen may be secreted out as peptides, proteins or metabolites into the supernatant. In *E. coli* and mammalian cells, previous studies found that glucose overflow metabolism resulted in acetate and lactate secretion into the medium.^33,34^ Algae can naturally secrete proteins like exozyme as well as fatty acid derivative compounds like diacylglyceryl-N,N,N-trimethylhomoserines (DGTS).^35,36^

Under nitrogen restricted conditions, biomass composition analysis indicated that the *i*CZ946-PAT5-10%AA genome-scale model could be applied to successfully limit the nitrate uptake rate while channeling the limiting element into synthesis of chlorophyll and other proteins. The goal was to retain sufficient chlorophyll and other protein content needed to maintain energetic and physiological processes critical to fatty acid production. Indeed, previous researchers found that degradation of chlorophyll decreases light energy conversion efficiency in red algae *Porphyridium cruerntum*.^37^ Further, in *Chlorella*, higher amounts of NADPH are required for lipid synthesis under nitrogen deprivation, but somewhat contradictory, photosynthesis from chlorophyll is the major source for NAPDH production.^38–40^ Using MMC to control the nitrate feed supply at reduced levels allowed the cells to continue to grow and accumulate biomass, including chlorophyll, while also synthesizing lipids, resulting in higher overall lipid and starch production rates as compared to the complete nitrogen deprivation case. Consequently, algal growth continued as a result of MMC, even under nitrogen restricted conditions from 261 to 549 h, which was the major fatty acid production stage, leading to higher yields of the fatty acid products compared to complete deprivation. Thus, maintaining sufficient chlorophyll level through limited nitrate feeding in the MMC-10%AA case allows the algae to support the energy demands needed for robust lipid synthesis in *C. vulgaris*. In addition, previous studies also found a correlation between lutein content and chlorophyll content in microalgae.^41^ The increase of lutein content in MMC-10%AA cultures also indicated the importance of optimizing nitrogen supply to sustain chlorophyll and lutein yield under nitrogen-limited conditions. To our knowledge, this is the first time researchers have applied a genome model to increase yields of both bioenergy precursors in the form of fatty acids and health products such as lutein in algal cultures systems under nitrogen-limited conditions.

Similar fatty acid profiles were observed for nitrogen restricted and nitrogen depleted conditions with C18:1 fatty acid as the dominant peak, as shown in Fig. 5a–c. Interestingly, this C18:1 fatty acid content dramatically decreased from ~15 to 18% DW for these nitrogen restricted conditions to approximately 0.65% DW under nitrogen replete conditions (Fig. S5). Previous studies found the same trend of increasing C18:1 content in *C. vulgaris* during a shift from nitrogen replete to nitrogen starvation conditions.^4,42^ The presence of a predominance of C18:1 fatty acid in all three cultures of this study (nitrogen depletion and nitrogen restriction) indicates that some levels of cellular response to nitrogen starvation were evident even for the two MMC groups in which the cells were fed with limited amounts of nitrate.

Previously, a study connected a kinetic model with a *C. reinhardtii* genome-scale model to optimize nitrate supply and light intensity for *D. salina* cultures.^43^ In this study, we expanded the capability of our multi-compartment *C. vulgaris* genome-scale models to control multiple nutrients, in this case glucose and nitrate, concurrently for *C. vulgaris* microalgal cultivation studies, including different biomass compositions that can vary as a result of the nutrient inputs. Previously, control has been implemented for algal fed-batch heterotrophic cultures using conventional methods to control glucose concentration within a certain range^44^ or kinetic models to optimize feeding rate.^25^ However, the previous model predictive methods used simple correlations to describe the kinetic interaction between substrate uptake rate and biomass build up rate. In this study, we have demonstrated how genomic information, metabolic networks and biomass compositions can be incorporated to build metabolic model control in order to provide a more precise understanding and control of heterotrophic algal cell cultures. In addition, previous studies observed that a high amount of glucose in the medium can negatively inhibit cell growth in two different *Chlorella* species.^44^ Our strategy successfully controlled glucose and nitrate at low concentration, which may be particularly useful for scaling up bioreactors in order to limit nutrient input costs and remediate glucose inhibition challenges.

Overall, this study demonstrates the power of building genome-scale metabolic models control for nutrient optimization with microbial algal cultures. As data collecting processes evolve, genome-scale models can be implemented into biomanufacturing in order to predict the specific amount of nutrients needed to support algal growth for different conditions and final process objectives as illustrated in supplemental Fig. S6. This systematic control strategy represents a potentially promising method for enhancing biofuel precursor production rates from microalgal cultures while controlling inputs in order to lower overall production costs of algal bioprocessing. A similar genome-based control approach involving restricted nutrient feeding could also be considered for optimizing productivities for other cell factories that generate valuable products during non-growth phases such as yeast production of organic acids^45^ and mammalian cell generation of recombinant proteins.^46^

## Methods

### Algal strain and cultivation conditions

Green microalgae *Chlorella vulgaris* UTEX 395 was obtained from the Culture Collection of Algae at the University of Texas at Austin and maintained on sterile agar plates (1.5% w-v) containing Bold’s Basal Medium (BBM). Liquid cultures were inoculated with a single colony in 12.5 mL of sterile BBM. Cells were transferred to 100 mL or 250 mL glass Fernbach flasks (New Jersey, USA) at 25 °C using BBM. Autotrophic cultures were grown with different nitrate concentration under fluorescence illumination (30 μE m^−2^ s^−1^). Heterotrophic cultures were grown with different glucose and nitrate concentration with 24 h dark. Nitrogen starvation cultures were grown under BBM without nitrate as nitrogen source. The growth of the cultures was monitored by measuring optical density (OD) at 750 nm. During the metabolic model control, specific amounts of 25 g/L nitrate or 20 g/L glucose were added manually into the cultures based on model prediction every 20–28 h. All the cultures were done in biological triplicate in this study.

### Measurement of algal biomass dry weight and FAME content

Liquid cultures were harvested using a high-speed centrifuge (Beckman J2–21, Baltimore, USA) at 4000 x g for 10 minutes. The pellets were stored at −80 °C and lyophilized for 24 h at −40 °C under freeze-dried machine.

FAME production followed the procedure provided by.^47^ FAMEs were analyzed using a Agilent’s Gas Chromatography (GC) system with discharge ionization detection equipped with a capillary column (Stabilwax-DA, 30 m 0.25 mm ID, film thickness 0.25 mm). GC inlet was set at 250 °C and the injections were in a volume of 1 μL. The temperature program started at 50 °C and then increased to 170 °C at a rate of 20 °C min^−1^, with a plateau for 1 min. After this plateau, the temperature increased from 170 °C to 220 °C at a rate of 4 °C min^−1^ and then kept constant for 14 minutes. The total analysis time was 35 minutes. Helium was used as carrier gas.

### Measurement of lutein yield, starch content, chlorophyll content and protein content

Lutein extraction followed the procedure provided by.^48^ The dried algae pellets (5–10 mg) were homogenized using a mortar and pestle with 4 mL extraction solvent, the mixture of dichloromethane (25%) and methanol (75%), for 2 min and 2 times. The extraction solution was centrifuged at 10000 x g for 10 min and kept in dark in −20 °C. The solution was filtered before HPLC analysis. The mobile phases are eluent A (dichloromethane: methanol: acetonitrile: water, 5.0:85.0:5.5:4.5, v/v) and eluent B (dichloromethane: methanol: acetonitrile: water, 25.0:28.0:42.5:4.5, v/v).

Starch content in dry biomass was analyzed using an assay kit (K-TSHK, Megazyme). Starch in dried algae pellets were hydrolyzed into glucose by α-amylase and amyloglucosidase. Then glucose was digested by enzyme hexokinase and glucose-6-phosphate dehydrogenase. After the reaction, the absorbance at 340 nm was measured.

Chlorophyll content followed procedure provided by.^49^ Chlorophyll in the dry algae pellets was extracted by DMSO and absorbance at 665 nm and 649 nm were measured.

Protein content followed procedure provided by.^4^ Dry Biomass was sonicated on ice for 4 °C for 30 s × 6 cycles. Lysates were centrifuged at 16,000 x *g* for 2 min and the supernatant were analyzed by bicinchoninic acid assay (BCA assay).

### Genome scale model simulation for nutrient control

The *i*CZ946 model was obtained from.^16^ Biomass composition, RNA-seq and proteomics data were collected previously under photoautotrophic (PA) conditions and heterotrophic (H) conditions.^4,50^ Six sample points were collected to build six different photoautotrophic models (PAT1-PAT6) and five samples were used to construct five different heterotrophic models respectively (HT1-HT5) as described in our previous publication.^16^ Data collected for all models, except PAT1 and HT1, were undertaken during nitrogen depletion conditions (Fig. S1). *i*CZ946-PAT1 model was applied for nutrient optimization in autotrophic conditions and *i*CZ946-HT1 model was used for nutrient optimization in heterotrophic conditions. The *i*CZ946-PAT5-10%AA and *i*CZ946-PAT5-2%AA models were built by changing amino acid composition in *i*CZ946-PAT5 model. The growth rate (μ) was constrained in the model and the objective function was changed to minimize nitrate uptake rate (F_N_) or glucose uptake rate (F_G_). Genome-scale model simulations were performed using the Gurobi Optimizer Version 5.6.3 (Gurobi Optimization Inc., Houston, Texas) solver in MATLAB (The MathWorks Inc., Natick, MA) with the COBRA Toolbox.^51^

## Supplementary information

reporting-summary

Supplementary information

## Data Availability

The data that support the findings of this study are available from the corresponding author upon reasonable request.
