# Supplementary material for: Utilizing genome-scale models to optimize nutrient supply for sustained algal growth and lipid productivity
Source: NPJ Syst Biol Appl. 2019 Sep 24;5:33. doi: 10.1038/s41540-019-0110-7 (PMC6760154; doi:10.1038/s41540-019-0110-7)
Supplement: Supplementary file 2 — Supplementary information [file 41540_2019_110_MOESM2_ESM.docx]

Supplementary Information

| Model | Condition | Biomass composition | Reference |
| --- | --- | --- | --- |
| iCZ946-PAT1 | Autotrophic  Nitrogen replete | 55% Amino acid, 16% Lipid, 26% Carbohydrate | Zuniga et al 2017 |
| iCZ946-PAT5 | Autotrophic  Nitrogen limited | 16% Amino acid, 47% Lipid, 36% Carbohydrate | Zuniga et al 2017 |
| iCZ946-HT1 | Heterotrophic  Nitrogen replete | 35% Amino acid, 27% Lipid, 34% Carbohydrate | Zuniga et al 2017 |
| iCZ946-PAT5-2%AA | Autotrophic  Nitrogen limited | 2% Amino acid, 47% Lipid, 36% Carbohydrate | This study |
| iCZ946-PAT5-10%AA | Autotrophic  Nitrogen limited | 10% Amino acid, 47% Lipid, 36% Carbohydrate | This study |

**Table S1** Summary of genome-scale models used in this work.

| Time points | 1 | 2 | 3 | 4 | 5 | 6 |
| --- | --- | --- | --- | --- | --- | --- |
| Compared with  NaNO_3_: 25 mg/L + 120% MMC | | |  |  |  |  |
| NaNO_3_: 25 mg/L + 80% MMC | P≤0.01 | P≤0.01 | P≤0.01 | P≤0.05 | P≤0.01 | P≤0.01 |
| NaNO_3_: 250 mg/L | ns | P≤0.05 | ns | P≤0.05 | ns | ns |
| NaNO_3_: 25 mg/L + 100% MMC | ns | ns | ns | ns | ns | ns |
| Compared with  NaNO_3_: 25 mg/L + 80% MMC | | |  |  |  |  |
| NaNO_3_: 250 mg/L | ns | P≤0.05 | P≤0.05 | P≤0.01 | P≤0.01 | P≤0.01 |
| NaNO_3_: 25 mg/L + 100% MMC | P≤0.05 | P≤0.01 | P≤0.01 | P≤0.01 | P≤0.01 | P≤0.01 |
| NaNO_3_: 25 mg/L + 120% MMC | P≤0.01 | P≤0.01 | P≤0.01 | P≤0.05 | P≤0.01 | P≤0.01 |

**Table S2** P-value at each time point in Fig. 1D. ns indicated the growth was not statistically significant,


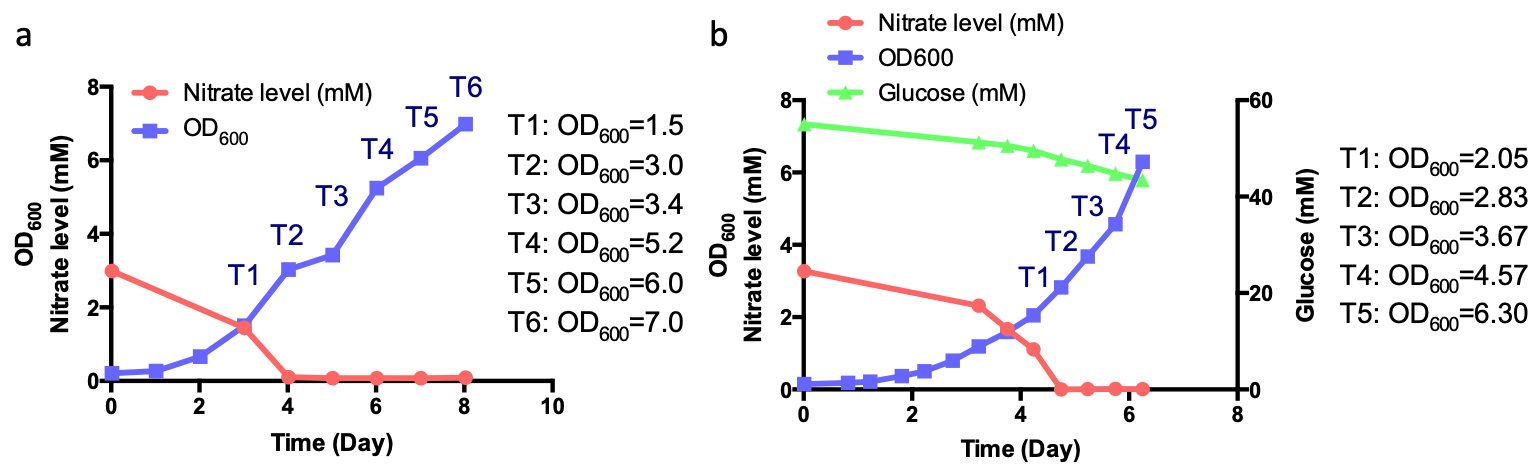


**Figure S1** Summary of the sample points collected to build *i*CZ946 model. **a** Autotrophic conditions; **b** Heterotrophic conditions


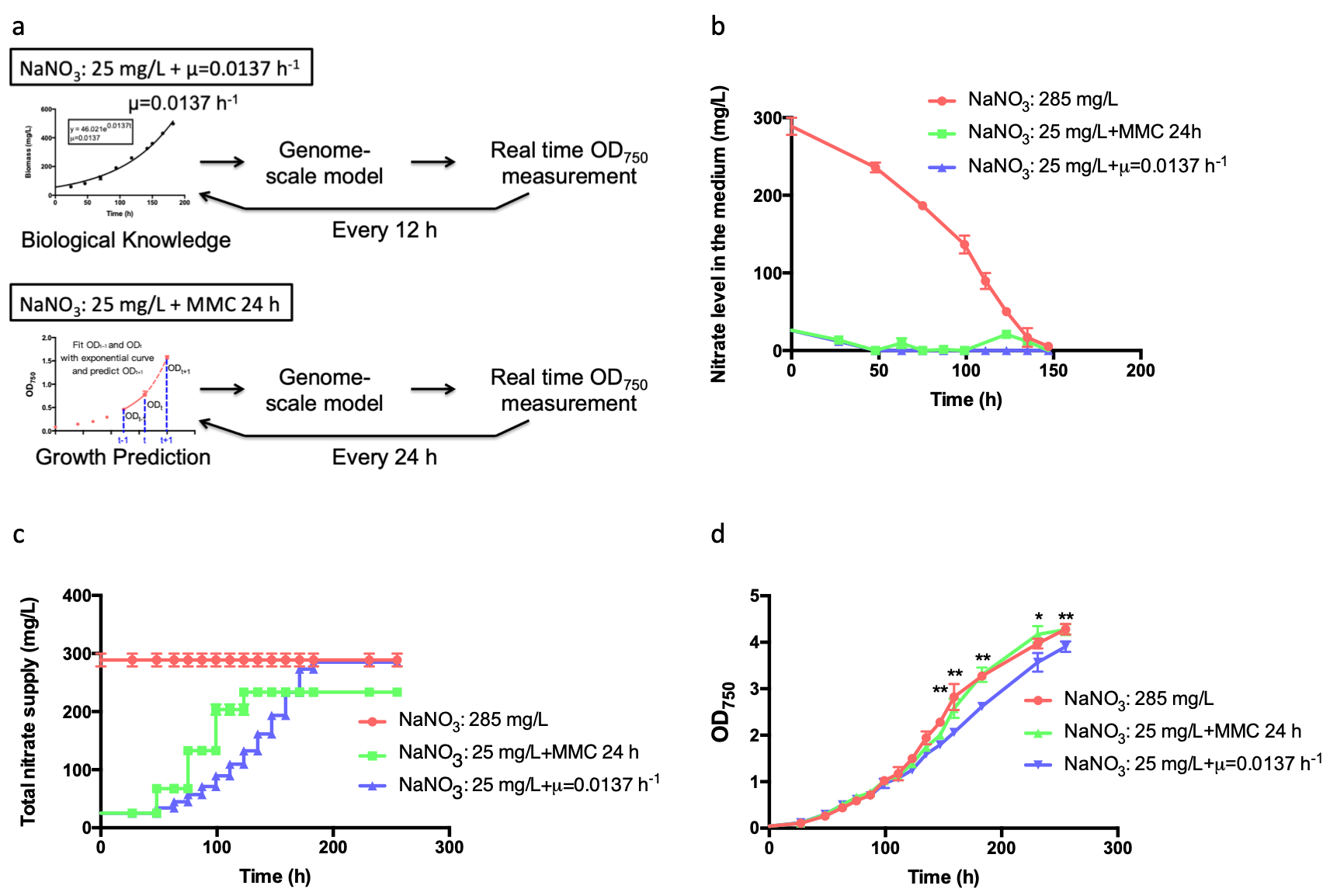


**Figure S2 a** Two different nutrient prediction methodology; **b** Nitrate level in the medium (mg/L); **c** Total nitrate supply (mg/L); **d** Growth rate (OD_750_). The data represents the mean ± SD for n=3. * P≤0.05 ** P≤0.01


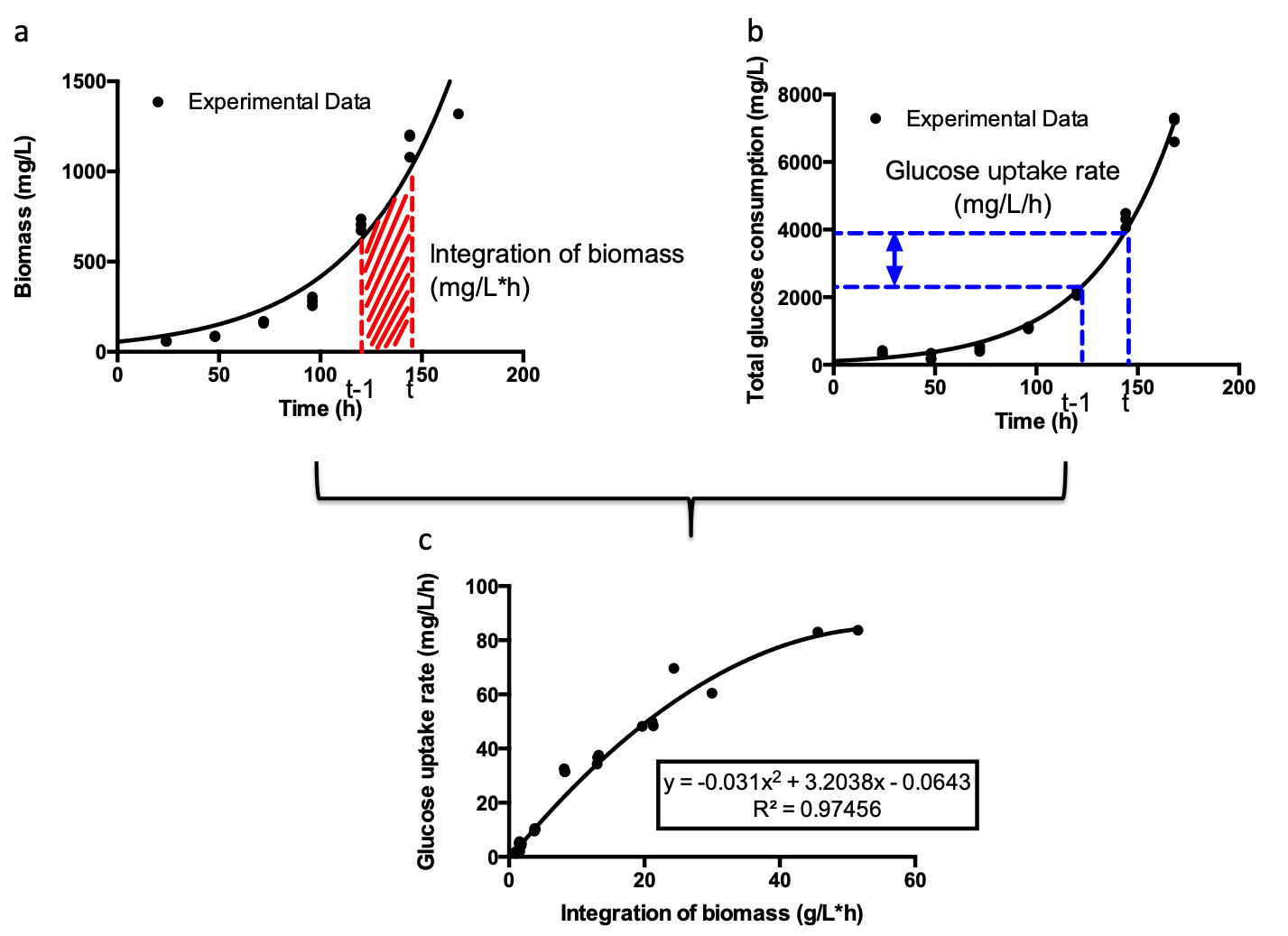


**Figure S3** Set up equation to describe the relationship between integration of biomass (g/L*h) and glucose uptake rate (mg/L/h). **a** Biomass accumulation (mg/L); **b** Total glucose consumption (mg/L); **c** Polynomial regression line to fit the data.


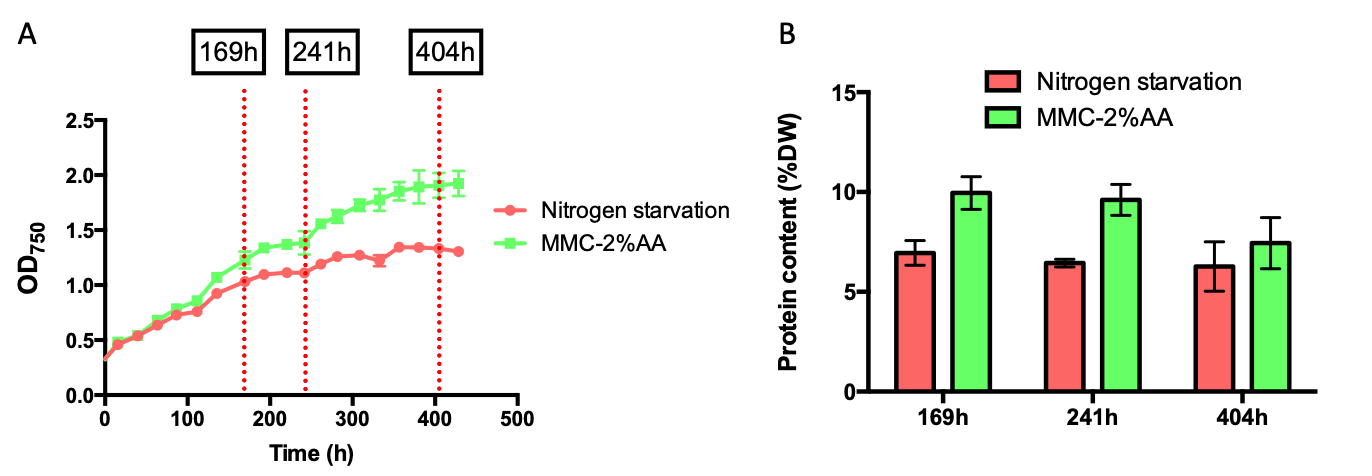


**Figure S4** Metabolic model control experiments under nitrogen starvation. (A) Growth rate (OD_750_) (B) Protein content (% DW). The data represents the mean ± SD for n=3.

**Figure S5** FAME content under nitrogen replete conditions, nitrogen starvation cultures at 549 h and two MMC cultures at 549 h. The data represents the mean ± SD for n=3 under nitrogen starvation (549 h), MMC-2%AA (549 h) and MMC-10%AA (549 h). n=2 under nitrogen replete conditions.


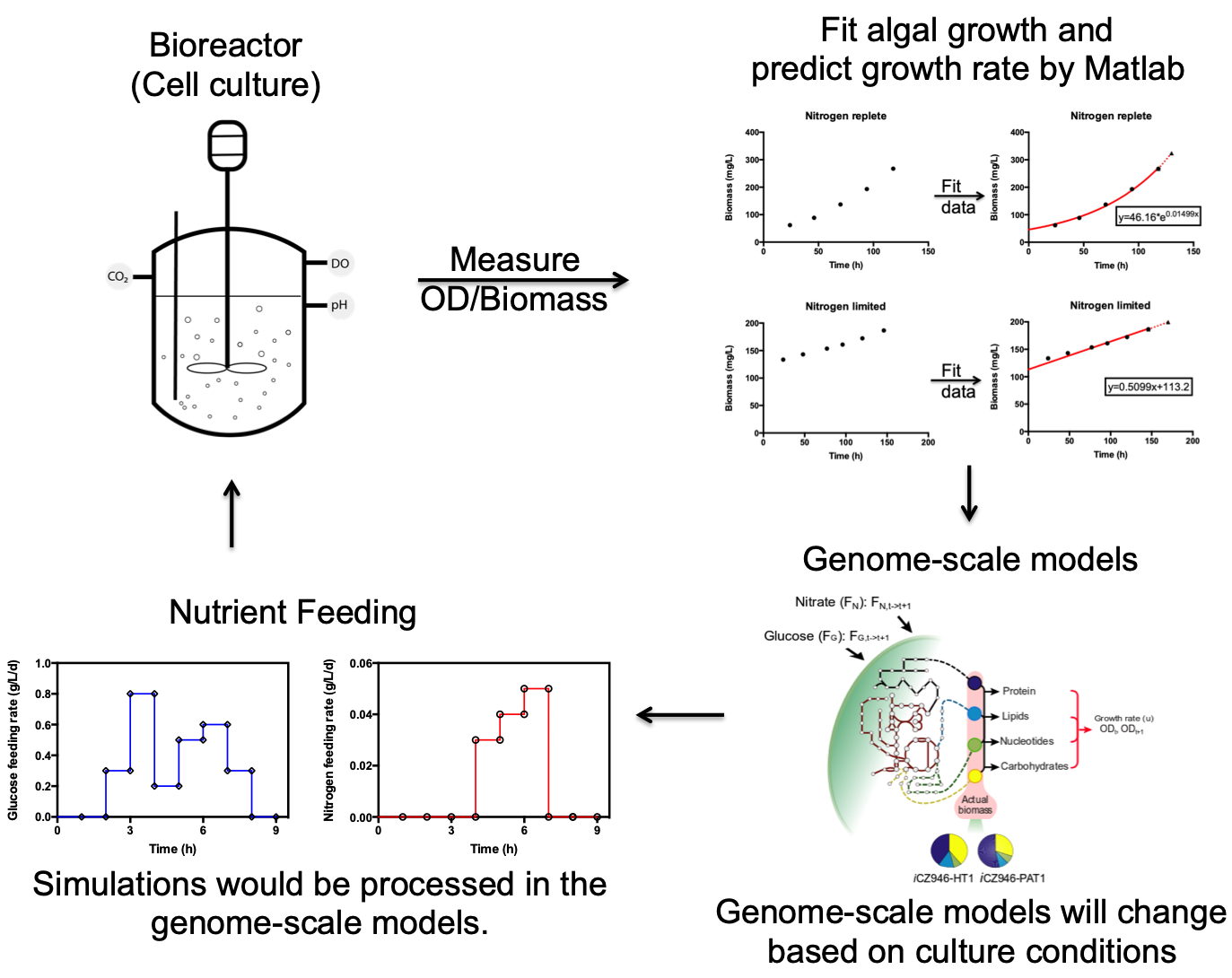


**Figure S6** Flowchart of genome-scale metabolic model control on cell culture bioprocessing
